# Way to efficient microbial paclitaxel mass production

**DOI:** 10.1016/j.synbio.2023.10.002

**Published:** 2023-10-19

**Authors:** Chenyue Li, Yanli Qi, Zhongke Sun, Mengwan Jiang, Chengwei Li

**Affiliations:** a School of Biological Engineering, Henan University of Technology, Zhengzhou, 450001, China; b Nanyang Institute of Medical Plant Technology and Industry, Nanyang, 473005, China; c School of Artificial Intelligence and Big Data, Henan University of Technology, Zhengzhou, 450001, China

**Keywords:** Paclitaxel, Microbial fermentation, Endophytes, Process optimization, Synthetic pathway

## Abstract

The microbial synthesis of paclitaxel is attractive for its short-cycle, cost-effectiveness, and sustainability. However, low paclitaxel productivity, depleted capacity during subculture and storage, and unclear biosynthesis mechanisms restrain industrial microbial synthesis. Along with the isolation of various paclitaxel-producing microorganisms and the development of versatile molecular tools, tremendous promises for microbial paclitaxel synthesis have become increasingly prominent. In this review, we summarize the progress of microbial synthesis of paclitaxel in recent years, focusing on paclitaxel-producing endophytes and representative engineering microorganism hosts that were used as chassis for paclitaxel precursor synthesis. Numerous wide-type microbes can manufacture paclitaxel, and fermentation process optimization and strain improvement can greatly enhance the productivity. Engineered microbes can efficiently synthesize precursors of paclitaxel by introducing exogenous synthetic pathway. Mining paclitaxel synthetic pathways and genetic manipulation of endophytes will accelerate the construction of microbial cell factories, indefinitely contributing to paclitaxel mass production by microbes. This review emphasizes the potential and provides solutions for efficient microbial paclitaxel mass production.

## Highlights

• Paclitaxel-producing microbes have the potential for paclitaxel mass production.
• Process optimization will promote microbial productivity and stability.
• Rewiring synthetic pathway is feasible for paclitaxel heterogeneous production.
• Engineered endophytic microorganisms are promising paclitaxel microbial cell factories.

## Graphical abstract

**Figure:**
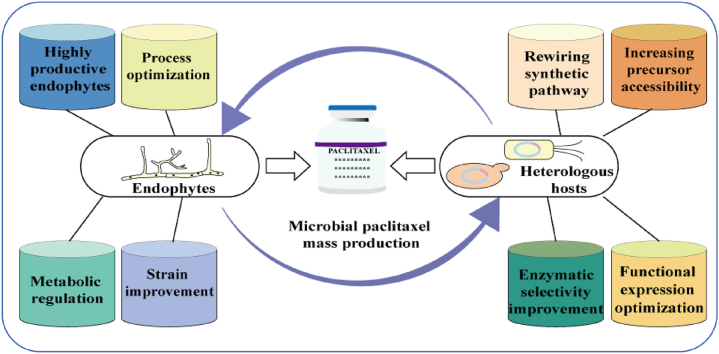

## 1 Introduction

As an effective anticancer drug originally extracted from *Taxus* plants, paclitaxel still cannot meet clinical demand due to the low yield and high cost in production. Compared with chemical synthesis and suspension culture of *Taxus* cells, microbial paclitaxel production has advantages of short production cycle, higher cost-benefit, and easier manipulation [1]. Generally, improving microbial paclitaxel synthesis needs selecting highly productive and stable paclitaxel-producing microorganisms, constructing optimal microbial chassis, and optimizing fermentation process [2,3].

In recent years, different genera of paclitaxel-producing microorganisms have been reported, which are potential candidate hosts for paclitaxel synthesis [4,5]. However, microbial paclitaxel productivity is generally very low. Screening novel microbes originating from different sources has expanded the diversity of paclitaxel-producing microorganisms, among which some possess yield of paclitaxel up to 1 mg/L [6,7]. Commercial scale-up of microbial paclitaxel requires further evaluation of the productivity stability of these high-yielding microorganisms. Unfortunately, attenuation of the metabolic capacity during subculture and storage has been found in many kinds of paclitaxel-producing microbes, and the machinery of this degeneration is still unclear, resulting a great distance to industrialization [8,9]. Thus, selecting high-yield strains, rewiring the synthetic pathway, and establishing a systematic and high-efficient optimization strategy are necessary to improve microbial paclitaxel synthesis [10,11]. However, integral paclitaxel metabolic pathway is still unknown in endophytes, and only partially elucidated in *Taxus* plants, limiting the metabolic optimization of paclitaxel synthetic pathway in paclitaxel-producing microbes [12]. D*e novo* synthesis of paclitaxel using microbial chassis is a fascinating approach, despite the entire synthetic pathway refactoring cannot be achieved yet [13]. Model microbial chassis such as *Escherichia coli*, *Saccharomyces cerevisiae*, and *Bacillus subtilis* often have distinct advantages [13,14]. Microbial expression systems for the biosynthesis of prophase intermediates involving in paclitaxel synthesis have been constructed [15]. These heterologous production systems have potential for microbial paclitaxel supplement due to high efficiency, easy-handle and low costs [16]. However, the problem of poor functionality of CYPs genes and low titer of target products has not been fundamentally fixed [14].

In this review, we discuss recent advances in paclitaxel synthesis by paclitaxel-producing endophytes and the precursors of paclitaxel by recombinant microorganisms. On one side, with the isolation and identification of paclitaxel-producing endophytes from various origins, combined with fermentation process optimization and strain improvement, paclitaxel productivity has been considerably strengthened. On the other side, we address how to explore promising industrial microorganisms and endophytes as cell factories to enhance paclitaxel and its precursors production. Depending on tremendous advancements in aforementioned topics, microbial synthesis shows great potential for efficient and sustainable mass production of paclitaxel.

## 2 Screening wide-type paclitaxel-producing microorganisms

The discovery of the paclitaxel-producing endophytic *Taxomyces andreanae* in *Taxus brevifolia* opened the door to explore plant endophytic for paclitaxel synthesis [4]. After that, tremendous studies reported the isolation of paclitaxel-producing microorganisms from versatile niches. Table 1 listed some representative paclitaxel-producing microorganisms, with three highlighted isolates producing more than 1 mg/L of paclitaxel, making them valuable resources for potential paclitaxel mass production.

**Table 1:** some representative microorganisms producing paclitaxel.

Genera	Endophyte	Host	Paclitaxel Yield(μg/L)	Methods of Optimization	Final yield (μg/L)	Reference
***Alternaria***	***Alternaria alternata* MF5**	***Taxus***	**5700**	**-**	**-**	[19]
	*Alternaria brassicicola* MVR1	*Terminalia arjuna*	140.8			[24]
	*Alternaria tenuissima* TER995	*Taxus arjuna*	37.92	Bioprocess optimization	124.32	[31]
*Annulohypoxylon*	*Annulohypoxylon* sp.	*Taxus wallichiana* Zucc.	282.05	–	–	[32]
	*Aspergillus flavipes* ATCC 24,487	Rhizosphere	185	Addition of fluconazole (1.0 μg/ml) or *P. gracilior* leaf (0.5 g)	320/210	[33]
*Aspergillus*	*Aspergillus flavus* MW485934.1	jojoba	88.6	Bioprocess optimization and γ-irradiation	375.9	[34]
	*Aspergillus fumigatus* TXD105	*Tsxus distichum*	84.41	Bioprocess optimization	307.03	[31]
	***Aspergillus fumigatus***	***Taxus* sp.**	**1590**	**-**	**-**	[6]
	*Aspergillus oryzae*	*Tarenna asiatica*	95.04	–	–	[35]
	*Aspergillus terreus* EFB108	*Podocarpus gracilior*	114.2	Physicochemical optimization and addition of surface-sterilized *P. gracilior* leaves	432	[23]
*Cladosporium*	*Cladosporium cladosporioides* MD2	*Taxus media*	800	–	–	[36]
	*Cladosporium sphaerospermum* AUMC 6896	Clover leaf weevil	3.732	Adding ammonium acetate (20 mg/L)	30.365	[37]
*Epicoccum*	*E. nigrum* TXB502	*Taxus baccata*	61.35	Bioprocess optimization, γ-irradiation and immobilization	1364.63	[38]
	*Fusarium redolens*	*Taxus brevifolia*	70	Bioprocess optimization	198	[39]
*Fusarium.*	*Fusarium solani* Tax-3	*Taxus chinensis*	163.35	–	–	[18]
*Grammothele*	*Grammothele lineata* SDL-CO-2015-1	*Corchorus olitorius*	382.2	–	–	[22]
*Lasiodiplodia*	*Lasiodiplodia theobromae* SKJM1101	*Piper nigrum*	247	–	–	[40]
*Metarhizium*	*Metarhizium anisopliae* H-27	*Taxus chinensis*	846.1	–	–	[17]
*Metarizium*	*Metarizium anisopliae* AUMC 5130	clover leaf weevil	0.0023	Adding both ammonium acetate (20 mg/L) and Salicylic acid (90 mg/L)	116.373	[37]
*Nodulisporium*	*Nodulisporium sylviform* HQD_33_	*Taxus cuspidate*	51.06–125.70	Strain improving	516.37	[41]
*Penicillium*	*Penicillium chrysogenum* R16	*Glycin max*	170	Bioprocess optimization	250	[26]
	*Penicillium polonicum* AUMC14487	*Ginko biloba*	90.53	Bioprocess optimization and γ-irradiation	401.2	[25]
***Pestalotiopsis***	***Pestalotiopsis hainanensis***	***Ailuropoda melanoleuca***	**1466.87**	**-**	**-**	[7]
*Pestalotiopsis*	*Pestalotiopsis microspora*	*Taxodium mucronatum*	283.11	Addition of Salicylic acid(300 μM)	625.47	[42]
*Phoma*	*Phoma medicaginis*	*Taxus wallichiana* var. *mairei*	1215	–	–	[43]

### 2.1 Paclitaxel-producing microorganisms from *Taxus*

Several endophytic microbes of *Taxus* have been shown to serve as potential sources of bioactive paclitaxel. *Metarhizium anisopliae* H-27 and *Fusarium solani* Tax-3, both originated from *Taxus chinensis* tissue, produced paclitaxel up to 846.1 μg/L and 163.35 μg/L, respectively [17,18]. Furthermore, another example that *Alternaria alternata* MF5, derived from the bark of female *Taxus* yew, can yield paclitaxel up to 5.7 mg/L after 60 h of fermentation, which is the fastest endophyte in terms of paclitaxel accumulation rate [19]. The high yield of up to 1.60 mg/L paclitaxel from *Aspergillus fumigatus* KU-837249 was reported, albeit inconsistent data [6]. However, no further reports validate its reproducibility and scalability during repeated subculturing. The underlying reason may be its poor purity or inability to stably maintain desired yield [20,21]. Besides, there is not yet distinct molecular mechanisms of paclitaxel biosynthesis in this strain. These limitations are still a nonnegligible hurdle for achieving its industrial availability.

### 2.2 Paclitaxel-producing microorganisms from other sources

Many paclitaxel-producing microbes isolated from *Taxus* tissues are also presented in other niches, demonstrating that paclitaxel-producing microorganisms may have a wider distribution [22]. Indeed, paclitaxel-producing endophytes isolated from non-*Taxus* medicinal plants are becoming increasingly common [23,24]. The discovery of paclitaxel-producing endophytes from other plant species have further expanded the biological diversity. Endophytes *Grammothele lineata* SDL-CO-2015-1 isolated from the herb Jut is the first paclitaxel producer that belongs to the Basidiomycota phylum, revealing that this phylum may harbor other potential candidates for paclitaxel production [22]. Paclitaxel-producing endophyte *Penicillium polonicum* from non-*Taxus* sources shows antimicrobial and antiproliferative properties similar to authentic paclitaxel and retains 80 % paclitaxel yield after 5 generations of subculturing [25]. Moreover, some paclitaxel-producing microorganisms from non-plants sources also represent a high potential for these strains to be promising alternatives. For example, the maximum amounts of paclitaxel-producing *Penicillium chrysogenum* from the rhizosphere region of *Glycine max* could reach 250 μg/L [26]. In addition, a soil-dwelling saprophyte was identified as *Aspergillus flavipes*, and no significant paclitaxel productivity decline was found after 10th subculturing [27]. Interestingly, *Pestalotiopsis hainanensis* isolated from animal provides higher paclitaxel yield than endophytic *Pestalotiopsis* fungi from other resources [7]. Exploring various paclitaxel-producing endophytic, parasitic and saprophytic microbes may overcome the limitations of traditional host organisms and provide a robust production platform.

The attenuation of paclitaxel-producing potency among endophytes is a common physiological phenomenon. It is reported that the initial paclitaxel yield of *C. cladosporioides* MD2 isolated from *Taxus* was 800 μg/L, contrasting to only 5–7 μg/L after 5 years of storage and subculturing [28]. The paclitaxel yield (350 ng/μL) of *Periconia* sp. isolated from *Torreya grandifolia* plant decreases to 118 ng/μL after three rounds of subculturing [21]. This decline in productivity implies that endophytes may have unique paclitaxel synthetic machinery that profoundly differs from *Taxus* [29]. Mining paclitaxel synthesis pathway, maintaining stable producing potency and obtaining higher productivity in endophytic microbe remains prerequisites for the large-scale production of paclitaxel [30]. Searching for potential microbes with stable molecular machinery system is a promising avenue for microbial paclitaxel production. Further research indefinitely needed to do is evaluation of their production stability, elucidate paclitaxel biosynthesis machinery and seek effective strategies to restore the productivity and develop high-effective microbial-based bioprocess for the scale-up of microbial paclitaxel.

## 3 Strategies to improve paclitaxel production in wide-type endophytes

### 3.1 Optimizing nutrients supply and fermentation parameters

**Fig. 1:**
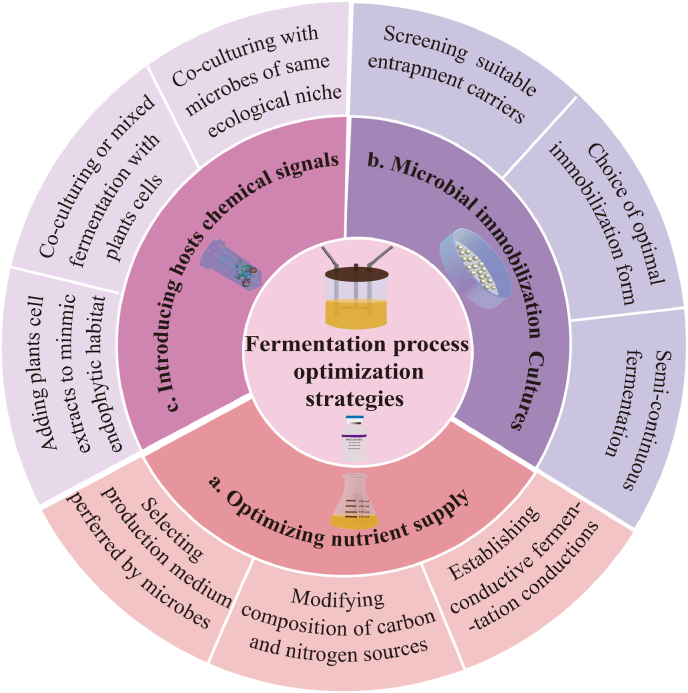
Strategies for fermentation optimization during paclitaxel production by endophytes. a. Optimizing nutrient supply to coordinate microbial growth and secondary metabolism. b. Applying microbial immobilization cultures to improve the overall efficiency of the production process. c. Introducing chemical signals from hosts to trigger the expression of genes in paclitaxel biosynthetic pathway.

Optimizing the nutrient composition of the culture medium could improve the growth of endophytes and the biosynthesis of paclitaxel (Fig. 1a). For example, the highest paclitaxel accumulation in *A. fumigatus* was reached in M1D media, while *Alternaria tenuissima* gave the highest paclitaxel yield in FBM media [31]. The type and ratio of different nutrients in medium also affect paclitaxel yield. The stimulatory effect of certain carbon and nitrogen sources on the synthesis of paclitaxel was widely recognized in endophytes, e.g., sucrose and ammonium nitrate are the optimal carbon and nitrogen sources for paclitaxel production by *A. fumigatus* TSD105 and *Fusarium redolen* [39,44]. Replacing sucrose in M1D medium with xylose as the carbon source resulted in induction of paclitaxel biosynthesis by endophyte *Aspergillus terreus* [44]. Moreover, adding solutes such as potassium chloride also showed stimulatory effect on paclitaxel accumulation by *Paraconiothyrium variabile* and *Epicoccum nigrum* [45].

Same to other secondary metabolites, the accumulation of paclitaxel is influenced by fermentation models and parameters. Cost-effective fermentation process is indispensable for its scaling up. It was found that solid-state fermentation systems gave higher concentrations of paclitaxel than liquid fermentation systems for endophytic *Nigrospora* sp [46]. Optimizing aeration and/or agitation rate to coordinate microbial growth and paclitaxel synthesis is fundamental in liquid fermentation [26]. The optimal agitation rate required to maximize paclitaxel production varied among microorganisms, with *A. fumigatus* and *A. tenuissima* producing the maximum at 120 rpm, whereas 150 rpm was the favorable speed for *A. fumigatus* TMS-26 [31]. Moreover, applying semi-continuous processing could greatly enhance fermentation productivity. El-Sayed et al. reported considerable yield enhancement after six cycles of semi-continuous fermentation of *A. fumigatus* TXD105-GM6 and *A. tenuissima* TER995-GM3 [47]. In all, as indicated in Table 1, the yield of paclitaxel by endophytes can be significantly increased by optimizing nutrients supply and fermentation conditions.

### 3.2 Immobilizing microbial cells

Microbial immobilization allows physical separation of cell and metabolite, also enables high-density enrichment and reuse of cells. Therefore, it is particularly useful for mass production of growth-inhibiting compounds like paclitaxel. In fact, immobilization technology has been reported to increase paclitaxel output by *Taxus* cells [48]. Entrapment carriers such as calcium alginate, gelatin, and others have been investigated to immobilize endophytes, achieving high-density enrichment of microbes and increase of paclitaxel output (Fig. 1b) [38,48]. Calcium alginate was considered the most suitable entrapment carrier for immobilizing *Aspergillus fumigatus* and *Alternaria tenuissima* mycelia and spores of mutant strains, as remarkable improvement of paclitaxel yields was achieved over free-cell cultures [48].

### 3.3 Introducing chemical signals from host plants

Unfortunately, recent findings suggested that the expression of paclitaxel biosynthetic gene cluster in endophytes may be dependent on chemical signals from the host plant and that artificial media cannot provide this specific microenvironment [49]. To overcome this limitation, one approach is adding plant extracts to mimic the habitat of endophytes and provide the necessary stimulus to microbes (Fig. 1c). For instance, adding gibberellic acid and cell extracts of *Corylus avellana* enhances paclitaxel yield by 11.5-fold in *E. nigrum* strain YEF_2_ [49]. Likewise, significant increase could be induced by supplementing plant wood or bark extracts for endophytic fungus *Paraconiothyrium* SSM001 [50]. Another approach is co-culture or mixed fermentation with plant cells. A 136.6-fold increase in paclitaxel output was achieved when co-culturing *E. nigrum* mycelium with host plants than endophytes fermented alone [51].

Additionally, competition for living space and trophic interactions between different endophytes of the same ecological niche trigger paclitaxel biosynthesis [52]. It was reported that co-culturing *Taxus* plants with only one resident fungus led to a 2.7-fold increase in paclitaxel output, while a 7.8-fold increase achieved by co-culture with two resident fungi [50]. The endophytic *B. subtilis* is a potent bacterial elicitor, and its intimate interaction with *A. flavipes* strongly triggered paclitaxel biosynthesis through chromatin remodeling [11]. In fact, mimic the close association among microorganisms by coculturing has been widely applied into natural products research. Coculture endophytic fungal *Fusarium tricinctum* originated from *Eichhornia crassipes* with *Bacillus subtilis* or *Streptomyces lividans* contributes to the biosynthesis of some novel valuable secondary metabolites, which has not been detected in monoculture [53]. This endophyte-plant or endophyte-endophyte interaction phenomenon is a kind of interspecies communication established for survive and function in their distinct ecological niches during evolution, though no clear understanding of this communication machinery [21,54]. One possible explanation is they serve as signals or transcriptional factors to activate the expression of orphan-gene involved in paclitaxel biosynthesis [55].

### 3.4 Regulating and maintaining metabolic balance

**Fig. 2:**
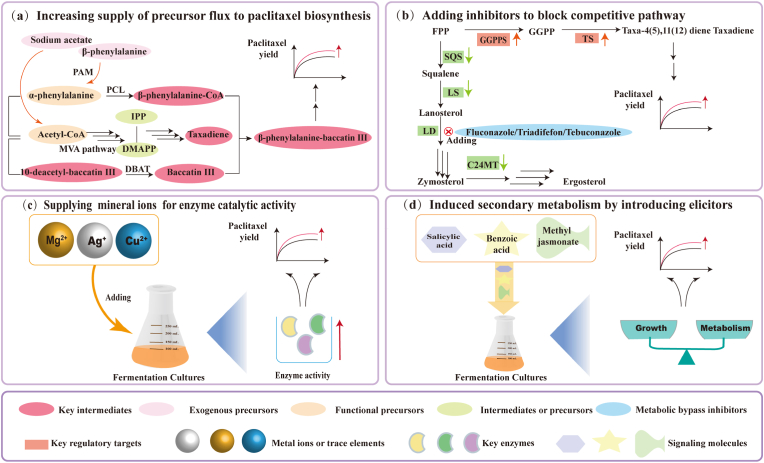
Solutions for metabolic regulation during paclitaxel synthesis by endophytes. (a) Increasing supply of precursor to facilitate the move of metabolic fluxes to paclitaxel. PAM, phenylalanine aminomutase; PCL, phenylalanine-CoA ligase; DBAT, 10-deacetybaccatin Ⅲ-10-O-acetytransferase; IPP, isopentenyl pyrophosphate; DMAPP, dimethylallyl pyrophosphate. (b) Adding metabolic bypass inhibitors to block competitive branches. TS, taxadiene synthase; GGPPS, geranylgeranyl diphosphate synthase; SQS, squalene synthase; LS, lanosterol synthase; LD, lanosterol demethylase; C24MT, C-24 methyltransferase. (c) Supplying mineral ions or trace elements to improve enzyme catalytic activity. (d) Introducing elicitors to regulate growth and metabolism balance.

Regulation of metabolic balance by exogenous growth factors and chemical substances is often important for efficient metabolite accumulation. Indeed, the shortage of precursors is one of the barriers to the massive production of paclitaxel in microorganisms (Fig. 2a). For instance, feeding common precursor isopentenyl pyrophosphate (IPP) and geranylgeranyl diphosphate (GGPP) to endophyte *Paraconiothyrium* SSM001 could improve paclitaxel yield by 3-fold and 5-fold, respectively [56]. Sodium acetate, an effective precursor in the acetylation reaction of paclitaxel core skeleton, induces the output of paclitaxel in endophytic fungal *F. redolens* and *Aspergillus aculeatinus* Tax-6 [39,57]. Like in *Taxus* cells, phenylalanine may has a positive effect for paclitaxel biosynthesis in endophytes [58].

However, high concentrations of exogenous precursors may cause toxicity and some intermediates tend to flow to other metabolic bypasses. Therefore, it is necessary to block the shift of precursors to competing branches and facilitate the conversion of metabolic fluxes to paclitaxel. Adding metabolic inhibitors can be effective in blocking bypass pathways (Fig. 2b). Sterols synthesis inhibitors have negative effect on squalene synthase, thereby promoting accumulation of GGPP towards taxadiene synthesis [2]. For example, adding fluconazole increases the taxadiene synthase (TDS) activity of *A. flavipes* and *A. terreus*, and the yield of paclitaxel was increased by 1.8-fold and 1.2-fold, respectively [59]. Moreover, fluconazole induces the expression of transcription factor *pbcR*, leading to increased paclitaxel production in *A. flavipes* [33]. However, microbes have unique metabolic backgrounds and growth characteristics, none of desired positive effects was obtained upon fluconazole stimulation in *A. terreus* EFB108 [23].

It was also demonstrated that suitable concentrations of mineral ions and trace elements is essential for microbial growth and paclitaxel synthesis (Fig. 2c). For example, Ag ^+^ can increase paclitaxel output by endophytes *A. terreus*, and Cu^2+^ induces the expression and catalytic activity of key enzymes in the paclitaxel synthesis pathway [23,29]. Typically, the yield of paclitaxel by *A. aculeatinus* Tax-6 has 4-fold increase upon optimization of concentrations of CuSO_4_ and other inducers [29]. Moreover, low concentrations of Mg^2+^ could help to stabilize the structure of TS and facilitate the binding of GGPP substrate. Reports on paclitaxel biosynthesis have shown that supplementation with suitable concentration of Mg^2+^ can increase the titer of paclitaxel by 2.1-fold [2].

Plant defense hormones are believed to trigger biosynthesis of paclitaxel by *Taxus* plant cells [23]. The yield of paclitaxel by endophytes has also an enhancement upon addition of phytohormones (Fig. 2d). Salicylic acid is responsible for activating GGPP synthase (GGPPS) by increasing reactive oxygen species and fatty acid peroxidation of unsaturated fatty acids, which serves as an induction signal to paclitaxel synthesis [42]. Similarly, adding salicylic acid to cultures of *Cladosporium sphaerospermum* AUMC 6896 and *Metarizium anisopliae* AUMC 5130 significantly increased paclitaxel output [37]. Moreover, benzoic acid may have a beneficial role in enhancing paclitaxel production [50]; however, recent radiolabeling studies have negated its direct contribution [23]. In addition, no significant increase in paclitaxel production was observed in several endophytes upon treatment with Methyl jasmonate, another phytohormone that often favors paclitaxel biosynthesis [29].

## 4 Rewiring industrial microbial hosts for paclitaxel and its precursor production

### 4.1 Increasing the titer of taxadiene

**Fig. 3:**
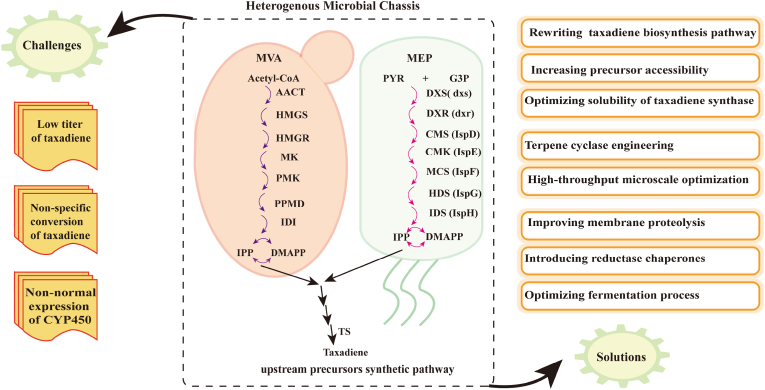
Challenges and solutions for metabolic engineering for taxadiene synthesis by heterogeneous host. The upstream paclitaxel biosynthetic pathway in heterogeneous microbial chassis is shown in dashed black box, which has distinction (the MVA and MEP pathways) in different expression systems. The main challenges (the left) and corresponding solutions (the right) have been listed. AACT, acetoacetyl-CoA thiolase; HMGS, 3-hydroxy-3-methylglutaryl-CoA synthase; HMGR, 3-hydroxy-3-methylglutaryl-CoA reductase; MK, mevalonate kinase; PMK, phosphomevalonate kinase; PPMD, mevalonate pyrophosphate decarboxylase; IDI, isopentenyl diphosphate isomerase; DXS, 1-deoxy-d-xylose-5-phosphate racemase; DXR, 1-deoxy-d-xylose-5-phosphate reductoisomerase; CMS, 4-pyrophosphocytidyl-2-C-methyl-D-erythritol synthase; CMK, 4-pyrophosphocytidyl-2-C-methyl-D-erythritol kinase; MCS, 2C-methyl-D-erythritol 2,4-cyclopyriophosphate synthase; HDS, 1-hydroxy-2-methyl-2-(E)-butenyl 4-pyrophosphate synthase; IDS, 1-hydroxy-2-methyl-2-butenyl 4-pyrophosphate reductase.

As mature microbial chassis, the fast-growing microbes such as *E. coli*, *S. cerevisiae* and *B. subtilis* can be engineered for potential paclitaxel production by introducing exogenous paclitaxel biosynthetic pathway [9,10]. Although the basic framework of paclitaxel synthesis pathway in plants has been determined, there are still a few genes haven't yet been characterized in plants [60]. These missing enzymes hindered heterogenous expression of complete paclitaxel biosynthetic pathway genes in engineering microbes [61]. At present, low level production of taxadiene, the precursor of paclitaxel was achieved by expressing IPP isomerase, GGPPS and TS [9,62]. Low taxadiene titer might be due to poor accessibility of precursors, high cytotoxicity of intermediates, low activity of key enzymes and presence of competing branches [62]. Current strategies to improve taxadiene titer include three main aspects: rewriting the taxadiene biosynthesis pathway, increasing precursor accessibility and optimizing the solubility of TS (Fig. 3).

Rewriting taxadiene synthetic pathway can remove the bottleneck and avoid growth inhibition caused by excessive accumulation of intermediates. Currently, modifying taxadiene biosynthetic pathway via optimizing the expression intensity and copy number of rate-limiting enzymes is a major approach [3]. By applying a systematic multivariate strategy to achieve the ideal expression equilibrium between the intrinsic upstream MEP pathway and the downstream IPP-to-taxadiene synthetic pathway in *E. coli*, the titer of taxadiene reached 1 g/L [62]. Also, using bidirectional promoters (BDPs) and optimizing expression time interval help a balanced state among different enzymes [15]. For example, the fine-tuning expression of GGPPS and TXS led to a 60-fold increase of taxadiene titer in yeast *Komagataella phaffii* after introduction of BDPs [63]. Similarly, the final titer of oxygenated taxanes reached 27 mg/L in engineering *E. coli* cell after metabolic balance [64]. In addition, the supplement of sufficient precursors is important for the scale-up of taxadiene biosynthesis in microbial hosts. The titer of taxadiene by *B. subtilis* could reach 17.8 mg/L by overexpressing TXS and optimizing the flux of GGPP [65]. Generally, low expression and poor solubility of TS exacerbated the difficulty in engineering taxadiene intermediate within microbial hosts. Researchers have demonstrated that the construction of stable and bioactive fusion protein of TS and GGPPS decreased the physical distance between the substrate GGPP and TS, leading to an elevated titer of taxadiene to 93.5 mg/L [57]. The length of TS truncation, type of promoter and copy number of chromosomal genes also impact microbial paclitaxel biosynthesis [15]. Taxadiene had exceeded 120 mg/L from 20 mg/L via increasing gene copy number and fusing suitable solubility tags in shake flask and bioreactors in *S. cerevisiae* [66].

### 4.2 Boosting specific conversion of taxadiene

It was reported that only 10 % of taxadiene was converted to T5α-ol due to the broad product profile of CYP725A4, which is a key obstacle in engineering paclitaxel biosynthesis in heterologous hosts [67,68]. Recent studies demonstrated that the nonspecific product is mainly due to the non-selectivity of the epoxide intermediate rather than to T5αH itself [67]. Previous approaches demonstrate that co-expressing CYP725A with CRP from different sources as a fusion protein resulted in decreased levels of all products [69]. Biggs et al. also found that chimera linkages may damage the functional expression of CYP450 in bacterial systems [70]. Although the yield of byproducts was decreased upon co-expression with *Taxus-*originated cytochrome B5, an apparent absence of T5α-ol was observed [69]. Moreover, improving the product specificity of the early synthetic pathway may favor alleviating this bottleneck. For example, the conversion of T5α-ol was improved by three approaches, terpene cyclase engineering, P450 engineering, and hydrolase screening [68]. TS engineering was proven to be feasible for improving CYP725A4 selectivity, then the yield of an alternative product taxa-4(20)-11(12)-dien was increased 2.4-fold (Fig. 3) [68]. Besides, the final titer of T5α-ol isomer and T5α-yl-acetate reached 19.2 mg/L and 3.7 mg/L by applying an interdisciplinary approach to optimize the oxidation and acetylation reactions of taxane in *S. cerevisiae* [71].

### 4.3 Improving the fitness of CYP450 family protein

CYP450 family enzymes involve in multi-step hydroxylation reactions of paclitaxel synthesis pathway, which are big roadblocks for *de novo* synthesis of paclitaxel in heterologous microbial cells [15]. The folding and post-translational modifications of these enzymes require multiple chaperones and a functional endosomal system, which are often absent or not fully functional in *E. coli* [62]. Similarly, in *S. cerevisiae*, the expression of CYP450 genes can be limited by insufficient supply of heme cofactors [62]. To overcome these limitations, the main solutions to strengthen CYP450 fitness are improving membrane proteolysis properties and introducing reductase chaperones (Fig. 3).

Although a 15000-fold improvement in taxadiene titer can be achieved by applying a multivariate modular metabolism engineering in *E. coli*, the titer of T5α-ol fell significantly upon the introduction of CYP725A4 [62]. Hence, modifying the hydrophobic N-terminus of membrane proteins and co-expressing of chaperonin are crucial for addressing the poor suitability of CYP450 in *E. coli* systems [72]. For example, five-fold enhancement of oxygenated diterpene can be achieved by optimizing the expression of CYP450 and CRP (reductase chaperones), and altering reductase partner interaction and N-terminal modifications of P450 and CRP in *E. coli* [70]. Compared with *E. coli*, yeast is a more amenable host for CYP450 expression due to its natural endomembrane system [64]. On one hand, function of CYP450 requires the incorporation of CRP for electron transfer [73]. For example, the optimal fusion of CYP725A4 and POR (cognate reductase) is beneficial for the formation of oxygenated taxanes in *S. cerevisiae,* due to improved activity of CYP725A4 [74]. On the other hand, enhancing the activity of rate-limiting enzymes with stronger promoters is a practicable method. GAL promoter is suitable for the construction of yeast cell factory. The GAL1 promoter drives combined expression of *Taxus* reductase and THY5a, leading to a 10-fold increase in taxadien-5a-ol in yeast [75]. In addition, riboregulated switchable feedback promoter (rSPF) regulated the expression of CYP725A4 and resulted in a 2.4-fold improvement of oxygenated taxanes in *E. coli* [76]. A modular co-culture system was also established between *E. coli* and *S. cerevisiae*, this mutualistic system combined the merits of different organisms, enabling more efficient and cost-effective production of oxygenated taxanes [77].

## 5 Engineering paclitaxel-producing endophytes for enhanced biosynthesis

### 5.1 Genetic manipulation of paclitaxel-producing endophytes

**Fig. 4:**
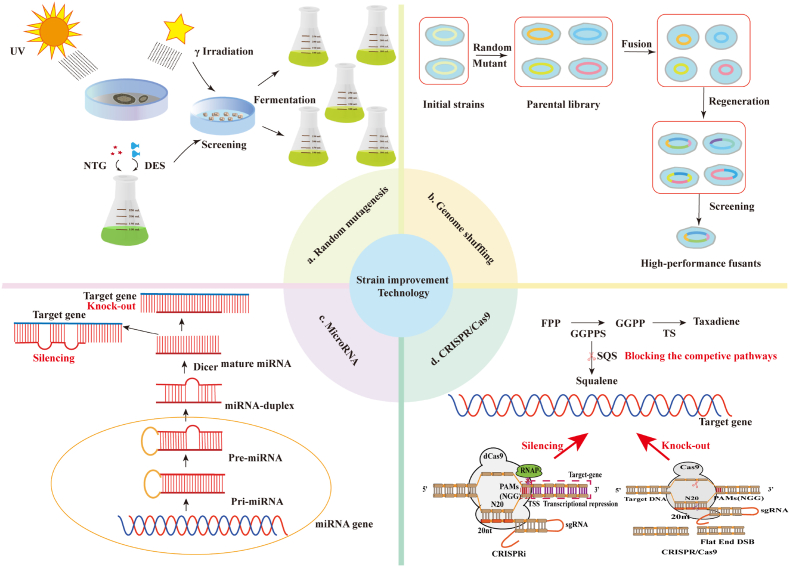
Genetic manipulation of paclitaxel-producing endophytes for strain improvement. a. Obtaining fine mutants by introducing chemical and physical mutagen. b. Genome shuffling through protoplast fusion, which introduces multiple mutations in the whole genome from multi-parents. c. Biogenesis of miRNA and gene silencing. d. Gene editing with the CRISPR/Cas9 technology and its application in paclitaxel biosynthesis regulation. The N20 sequence of the sgRNA recognizes the target gene, dCas9-mediated gene expression silencing has no DNA double-strand breaks, and Cas9 protein usually introduces flat-end DNA DSB (double-strand breaks) at the target gene site. NTG, nitrosoguanidine; UV, ultraviolet light; DES, diethyl sulfate; PAMs, protospacer adjacent motif; RNAP, RNA polymerase; FPP, farnesyl pyrophosphate; GGPP, Geranylgeranyl pyrophosphate; GGPPS, geranylgeranyl diphosphate synthase; TS, taxadiene synthase; SQS, squalene synthase.

Genetic manipulation of endophytes can be either irrational or rational targeted. Various high-yield mutants have been obtained through random mutagenesis using nitrosoguanidine (NTG), ultraviolet light (UV), diethyl sulfate (DES) and γ irradiation (Fig. 4) [78]. A 10-fold improvement of paclitaxel yield is found in *Fusarium maire* K178 upon UV irradiation and DES treatment [79]. Also, γ-irradiation results in a 2.42-fold enhancement of paclitaxel production in *A. fumigatus* TXD105 [80]. However, optimizing mutation conditions to coordinate growth and metabolism is also indispensable. For example, the best mutant dose of γ radiation is 0.75 KGy for *A. fumigatus* TXD105, but the highest yield of paclitaxel by *A. flavus* is obtained under 1.0 kGy [34,44]. Furthermore, genome shuffling allows multiple rounds of recursive fusion of protoplasts for rapid recombination in whole genome from multi-parents, making it a practical and effective strain improvement technique for obtaining high-performance fusants [81]. Using genome shuffling technology, Zhao et al. obtained three mutant strains with stable paclitaxel production and showed higher yield than the starting strain [41]. However, success of these irrational designs is largely depended on efficient high-throughput screening.

Further advancements in paclitaxel production in these microorganisms will be more likely obtained by applying synthetic biology technologies such as CRISPR/Cas9-mediated genome editing and RNA interference (RNAi). For example, RNAi and CRISPR/Cas9 technologies can be used to knockdown or knockout the rate-limiting enzyme lanosterol/squalene synthase in the sterol synthesis pathway, facilitate the metabolic flux to paclitaxel [10,82]. However, gene editing is relatively difficult in many paclitaxel-producing endophytes, especially filamentous fungi [82]. Recently, protoplast transformation mediated by electroshock or PEG/CaCl2 has successfully transferred exogenous plasmids containing resistance markers to the paclitaxel-producing endophyte EFY-21 [83]. *Agrobacterium*-mediated transformation (ATMT) is an effective method for gene transformation in certain endophytes. Bian et al. established an ATMT method for the genus *Alternaria* and validated the strength of heterologous promoters in *A. alternata* TPF6 [84]. Successful transformation of paclitaxel-producing fungi has enabled the introduction and optimization of heterologous taxadiene synthesis pathways, which are essential for improved paclitaxel biosynthesis. Moreover, developing high effective genetic manipulation system can indeed be essential. Recently, a feasible CRISPR/Cas9-mediated gene manipulation method is developed, which allows site-specific gene insertion, dual-locus mutations, and long DNA fragment deletions in endophytic fungus *Pestalotoiopsis ficiI* [85]. Genes involved in paclitaxel biosynthesis are regulated by different promoters and regulatory elements. Therefore, the transcription level of key genes could be optimized by modifying promoter strength, codon usage, and gene copy number. For instance, ectopic expression of GGPPS from *Taxus* has been proven feasible to enhance the accumulation of paclitaxel in endophytes [56]. In addition, little evidence for paclitaxel biosynthesis pathway was found in microbes, which hindered the directed evolution of paclitaxel pathway genes [5].

### 5.2 Deep mining of paclitaxel synthesis pathway in endophytes

Genome sequencing technologies and bioinformatics tools provide powerful support for identifying and characterizing paclitaxel biosynthetic mechanisms in endophytes. The key enzymes such as TS and 10-deacetylbacaccatin Ⅲ −10-O-acetyltransferase (DBAT) and C-13 phenylpropanoid side chain-CoA acyltransferase (BAPT) in *Taxus* have been identified, which provide a reference for mining microbial paclitaxel synthesis pathway and identification of involved candidate genes in endophytes [61]. On one side, the coding sequence can be used as reference to clone and characterize related enzymes in endophytes. For example, *dbat* gene and WRKY1 transcription factor in endophytes were amplified using primers based on corresponding genes in *T. cuspidate* [40]. Seven candidate genes in endophytic fungus *Penicillium aurantiogriseum* NRRL 62431 have also been identified, and the independent evolution of enzymes involved in paclitaxel biosynthesis is elucidated [86]. On the other side, comparative analysis of known paclitaxel biosynthesis genes in *Taxus* has provided insights into paclitaxel synthetic mechanism and evolution in microbes [12]. Partial potential synthesis pathway from GGPP to 10-DBAT was found in *C. cladosporioides* MD2, highlighting the diversity of biosynthetic routes in nature [28]. Moreover, comparative transcriptomes analysis of high-yielding mutant strains and starting strains found the expression change of T10βH [87].

However, the industrial utilization of microbial paclitaxel is still hindered by poor productivity, the undesired loss of production. The fundamental bottleneck is the lack of comprehensive understanding of the molecular machinery of paclitaxel biosynthesis, metabolic attenuation and transcript regulation network. One reason is that microenvironmental change may lead to low-level or even silence expression of critical functional genes [87]. Another is that different microbes may have evolved independently paclitaxel biosynthesis pathways, resulting in unannotation or detection of candidate genes due to low sequence identities [28,87]. Moreover, the substantial decrease in productivity during storage or subculture is accompanied by a decrease in the expression of key genes, which limits the in-depth exploration of the paclitaxel synthesis pathway in endophytes [8,28]. The complex attenuation machinery remains an unsolved mystery. A possible speculation is that the isolation of endophytes from hosts lead to disruption of native environmental interaction, resulting in the absence of chemical and biological stimulation, which are often difficult to mimic under *in vitro* conditions [54,88]. Furthermore, multiple subculturing may alter microbial nutritional requirements, resulted in the metabolic reprogramming and regulatory collapse of secondary and metabolite production in fungal cells [30,88]. Moreover, for the lipophilic nature of paclitaxel and its derivatives, their secretion may lead to cytotoxicity thereby inhibit microbial growth [74]. Overall, paclitaxel biosynthesis machinery in endophytes is still an unfilled gap, which is a prior condition for industrial scale-up of microbial paclitaxel.

## 6 Concluding remarks

Microbial synthesis provides a promising method for paclitaxel mass production. Isolation and identification of paclitaxel-producing endophytes bring the dawn of microbial synthesis of paclitaxel, and modification of paclitaxel synthesis pathway in microbes promotes the efficiency of microbial cell factories. Optimization of intermediate taxadiene, T5α-ol biosynthesis in heterologous hosts lays the foundation for the reconstitution and heterologous synthesis of the complete paclitaxel synthetic pathway. Future work can be performed from the following aspects: (1) developing high-throughput screening methods for the isolation of more novel, stable, and high-yield paclitaxel-producing microbes; (2) solving the problems of low yield and unstable productivity in microbes with effective strain improvement methods; (3) elucidating the molecular mechanism of paclitaxel synthesis by endophytes based on genomic, transcriptomic, and metabolomic studies; (4) reconstruction of high yield paclitaxel chassis with model microbial hosts or engineering paclitaxel-producing endophytes, using different synthetic biology techniques. Specially, system designing and synthetic engineering paclitaxel biosynthesis pathway in microbes can serve as a promising avenue for the high-level production of paclitaxel or its intermediates, including both industrial strains and paclitaxel-producing wide-type isolates [15]. These include 1) development of more efficient hosts chassis and genetic tools to accelerate construction; 2) characterization of key enzymes and editing of promoters as well as regulators to improve efficiency; and 3) optimization of fermentation bioprocess to ensure productivity.

## Authors’ contributions

LC and SZ contributed to the study conception and design. LY, QY, and JM performed literature search and data analysis. LY and QY wrote the first draft of the manuscript. QY and LC critically revised the work. All authors commented on previous versions of the manuscript and approved the final manuscript.

## Declaration of competing interest

The authors declare no competing interests.
